# Existence of *Pentatrichomonas hominis* in Tibetan Antelope (*Pantholops hodgsonii*)

**DOI:** 10.3389/fvets.2025.1493928

**Published:** 2025-02-05

**Authors:** Shuo Liu, Jing-Hao Li, Si-Yuan Qin, Jing Jiang, Zhen-Jun Wang, Tao Ma, Jun-Hui Zhu, Hong-Li Geng, Wei-Lan Yan, Nian-Yu Xue, Yan Tang, He-Ting Sun

**Affiliations:** ^1^College of Life Sciences, Changchun Sci-Tech University, Shuangyang, Jilin, China; ^2^College of Veterinary Medicine, Qingdao Agricultural University, Qingdao, Shandong, China; ^3^Center of Prevention and Control Biological Disaster, State Forestry and Grassland Administration, Shenyang, Liaoning, China; ^4^Key Laboratory of Zoonosis Research, Ministry of Education, College of Veterinary Medicine, Jilin University, Changchun, Jilin, China; ^5^College of Veterinary Medicine, Yangzhou University, Yangzhou, Jiangsu, China; ^6^College of Pharmacy, Guizhou University of Traditional Chinese Medicine, Guiyang, Guizhou, China

**Keywords:** *Pentatrichomonas hominis*, prevalence, Tibetan antelope (*Pantholops hodgsonii*), risk factors, China

## Abstract

**Introduction:**

*Pentatrichomonas hominis* is a conditional pathogen that parasitizes the intestines of vertebrates and has been detected in various wild animals. However, its infection rate in Tibetan antelopes has not been previously studied.

**Methods:**

In this study, 503 fecal samples from Tibetan antelopes were analyzed to determine the prevalence and molecular characteristics of *P. hominis*.

**Results:**

Results showed that 1.19% (6/503) of the samples tested positive, and although the prevalence was low, this finding underscores the importance of monitoring wild animals population as hosts of zoonotic pathogens. Additionally, the highest prevalence in Nima County (6.25%, 4/64), followed by Shenza County (2.44%, 2/82). No *P. hominis* was detected in samples from Shuanghu, Ruoqiang, Qiemo, and Qumarlêb Counties. Seasonally, the highest prevalence was recorded in autumn (1.42%, 6/423). Interestingly, *P. hominis* was only detected in 2020 (2%, 6/300), with no infections found in 2023 (0/50) or 2024 (0/153). Additionally, the phylogenetic analysis indicated that most islolates belonged to the CC1 genotype, with one representing a potential novel genotype.

**Discussion:**

This is the first s to report the presence of *P. hominis* in Tibetan antelopes, revealing that Tibetan antelopes may be a potential transmitter of zoontic *P. hominis*. These findings offer new insights into its epidemiology and contribute valuable data for Tibetan antelope conservation efforts.

## 1 Introduction

*Pentatrichomonas hominis* is an anaerobic, unicellular protozoan characterized by five anterior flagella and a single recurrent flagellum. It belongs to the genus *Trichomonadidae* within the phylum *Parabasalia* (1). *P. hominis* demonstrates broad host adaptability, commonly parasitizing the intestines of vertebrates, including dogs, cats, and pigs (2–4). Pathological reports have also confirmed its presence in the urinary tract of bulls (5). While *P. hominis* is typically associated with gastrointestinal disturbances in non-human primates, it is increasingly recognized as a potential pathogen in human gastrointestinal diseases (6, 7). Moreover, *P. hominis* has been linked to respiratory diseases, suggesting its involvement in a broader range of health conditions beyond gastrointestinal issues (8). These observations imply that *P. hominis* could act as a zoonotic pathogen, rather than merely existing as a commensal organism. In recent years, extensive epidemiological research has focused on *P. hominis* in a wide range of animals, such as owls, boa constrictors, Siberian tigers, cattle, sheep, and macaques (9–13). Notably, there is a related report in huamans in northern China. However, the prevalence of *P. hominis* in Tibetan antelopes remains unexplored.

The Tibetan antelope (*Pantholops hodgsonii*) is the only species within the genus *Pantholops* of the subfamily *Antilopinae*, and it is classified as a Class I protected animal in China (14). In 2016, the International Union for Conservation of Nature (IUCN) listed the Tibetan antelope as Near Threatened (15). Tibetan antelopes are known to harbor various pathogens, such as *Brucella*, *Blastocystis*, and *Cryptosporidium*. They may serve as an intermediate host, facilitating the transmission of these pathogens from livestock to humans (16–18). Therefore, this study hypothesized that Tibetan antelope could serve as a host for *P. hominis* to promote the transmission of zoonotic diseases.

This study aimed to assess the prevalence of *P. hominis*, phylogenetic analysis, and potential risk factors in Tibetan antelopes. Nested PCR was employed to detect *P. hominis* in fecal samples collected from Tibetan antelopes across Xinjiang, Tibet, and Qinghai. This study is the first to focus on the infection and prevalence of *P. hominis* in Tibetan antelopes, and provides new evidence of *P. hominis* infection in wild animals.

## 2 Materials and methods

### 2.1 Samples collection

**Figure 1:**
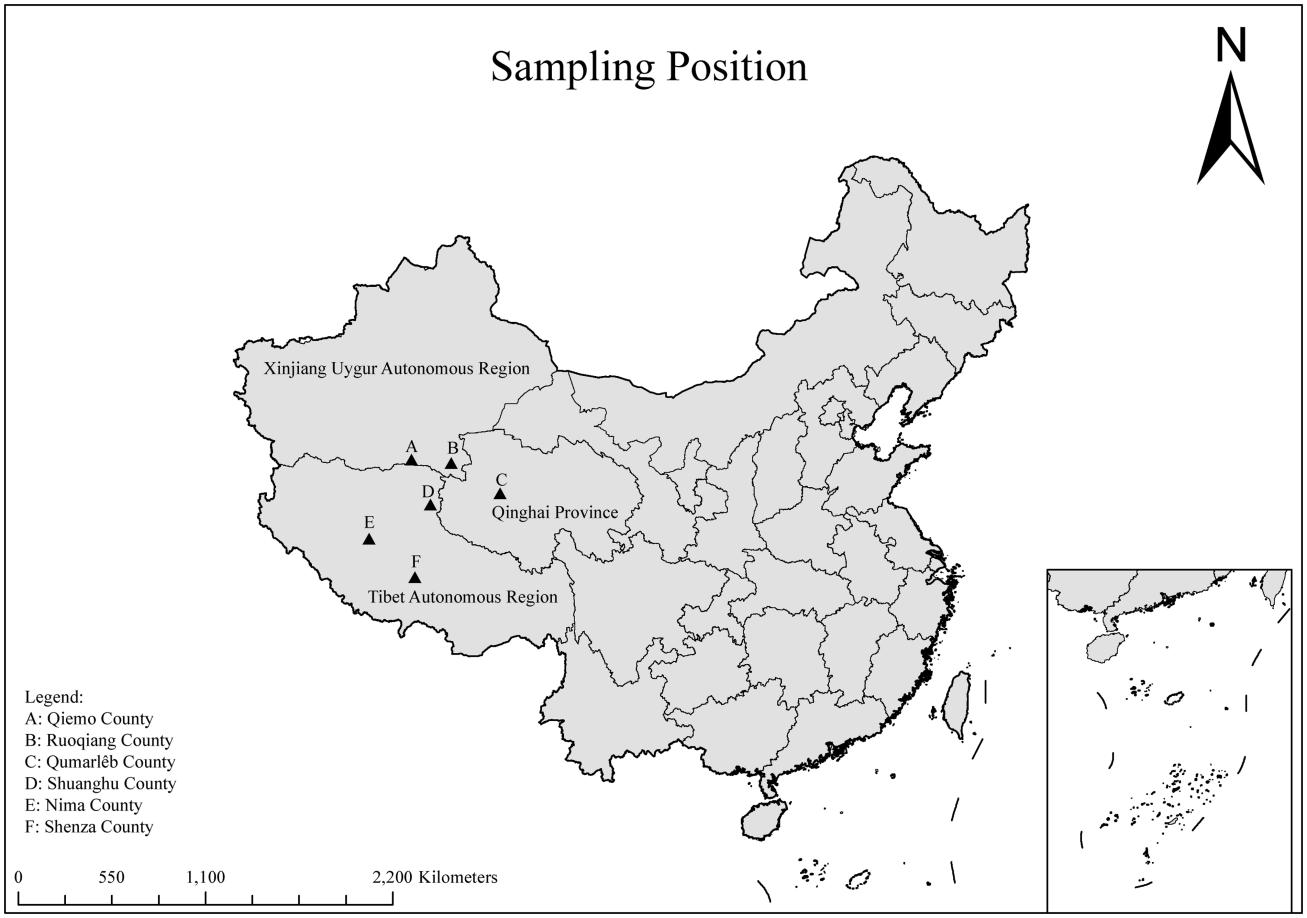
A map of the PR of China showing the sampling regions marked as triangles.

In September 2020 and from July 2023 to May 2024, a total of 503 fecal samples were collected from Tibetan antelopes in the Tibet Autonomous Region, Xinjiang Uygur Autonomous Region, and Qinghai Province, China (Figure 1). In the field, fresh fecal samples were randomly collected using disposable sterile gloves, immediately placed in sterile sample tubes, and stored in ice boxes. They were then transported to the laboratory under cold chain conditions and stored at −80°C.

### 2.2 DNA extraction and PCR amplification

For each sample, 200 mg of feces was placed in 2 mL centrifuge tubes containing 200 mg of glass beads, and 0.9% saline solution was added. The mixture was homogenized using a vortex mixer (JOANLAB, Zhejiang, China). Genomic DNA was extracted following the manufacturer’s protocol using the E.Z.N.A.® Stool DNA Kit (Omega Biotek, Inc., Norcross, GA, USA). The extracted DNA was stored at −20°C until PCR analysis. This study followed the guidelines and recommendations of the Animal Welfare Committee of Qingdao Agricultural University, China. The positivity rate of *P. hominis* was determined using nested PCR (19). In the first round of amplification, small subunit ribosomal RNA was amplified using forward primer FF (5’-GCGCCTGAGAGATAGCGACTA-3′) and reverse primer RR (5’-GGACCTGTTATTGCTACCCTCTTC-3′) to detect *Trichomonas* spp. A second round of amplification was performed using 2 μL of the first-round product, with forward primer HF (5’-TGTAAACGATGCCGACAGAG-3′) and reverse primer HR (5’-CAACACTGAAGCCAATGCGAGG-3′) to specifically detect *P. hominis* (339 bp). Both negative and positive controls were included in each PCR. For the second-round product, 5 μL was analyzed via electrophoresis on a 1.5% agarose gel and visualized under ultraviolet light.

### 2.3 Sequencing and phylogenetic analysis

PCR products positive for *P. hominis* were sent to Anhui General Biotech Co., Ltd. (Anhui, China) for sequencing. The resulting contigs from bidirectional sequencing were aligned with reference sequences in GenBank using BLAST.^1^ Phylogenetic trees were constructed using the neighbor-joining (NJ) method (20), with genetic distances calculated using the Kimura 2-parameter model in MEGA11 (21). The reliability of the phylogenetic trees was assessed with 1,000 bootstrap replicates. Representative nucleotide sequences were submitted to GenBank under accession numbers PQ276129-PQ276131.

### 2.4 Statistical analysis

The effects of sampling region (*x*1), sampling season (*x*2), and sampling year (*x*3) on the prevalence of *P. hominis* in Tibetan antelopes were analyzed using chi-square tests in the Statistical Analysis System (SAS, v9.0), with the best model selected using Fisher scoring. Logistic regression analysis was performed using Statistical Product and Service Solutions (SPSS, IBM Corp., Armonk, NY, USA), with 95% confidence intervals (95% CI) calculated. A *p*-value of less than 0.05 was considered statistically significant.

## 3 Results

### 3.1 Prevalence of *Pentatrichomonas hominis*

**Figure 2:**
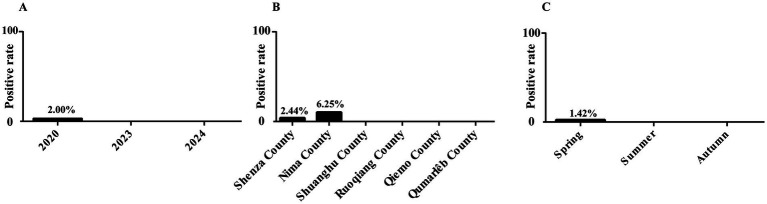
Infection rate of *P. homins* in Tibetan antelope under various factors. **(A)** Infection rate of *P. homins* in Tibetan antelope in different years. **(B)** Infection rate of *P. homins* in Tibetan antelope in different regions. **(C)** Infection rate of *P. homins* in Tibetan antelope in different seasons.

In this study, 6 out of 503 fecal samples were identified as positive for *P. hominis*, with an overall prevalence of 1.19% (6/503, 95% CI: 0.44–2.56). Nima County had the highest infection rate at 6.25% (4/64, 95% CI: 1.38–13.76), followed by Shenza County at 2.44% (2/82, 95% CI: 0.40–7.21). No *P. hominis* was detected in the remaining four counties. Seasonally, no infections were detected in spring or summer, with all positive cases found in autumn (1.42%, 6/423, 95% CI: 0.47–2.10). The prevalence of *P. hominis* was higher in 2020 (2%, 6/300, 95% CI: 0.67–3.95) compared to 2023 (0/50) and 2024 (0/153) (Table 1 and Figure 2).

**Table 1:** Prevalence of *Pentatrichomonas hominis* infection in Tibetan antelope (*Pantholops hodgsonii*) by various factors.

Variables	Categories	No. positive/total	Positive rate % (95% CI)	Heterogeneity
				*χ^2^*/*df*/*I^2^* (%)/*p*
Region	Shenza County	2/82	2.44 (0.40–7.21)	13.21/5/62.1/0.0215
	Nima County	4/64	6.25 (1.38–13.76)	
	Shuanghu County	0/204	0.00 (−)	
	Ruoqiang County	0/31	0.00 (−)	
	Qiemo County	0/30	0.00 (−)	
	Qumarlêb County	0/92	0.00 (−)	
Season	Autumn	6/423	1.42 (0.47–2.10)	0.61/2/0/0.7663
	Summer	0/30	0.00 (−)	
	Spring	0/50	0.00 (−)	
Year	2020	6/300	2.00 (0.67–3.95)	4.96/2/59.7/0.0837
	2023	0/50	0.00 (−)	
	2024	0/153	0.00 (−)	
Total		6/503	1.19 (0.44–2.56)	-

### 3.2 Risk factors

Logistic regression analysis using the Fisher scoring method was performed to assess the influence of sampling region, season, and year on *P. hominis* prevalence. The final model did not retain any of these factors, suggesting that sampling region, season, and year had no statistically significant impact on the infection rate of *P. hominis*.

### 3.3 Phylogenetic analysis

**Figure 3:**
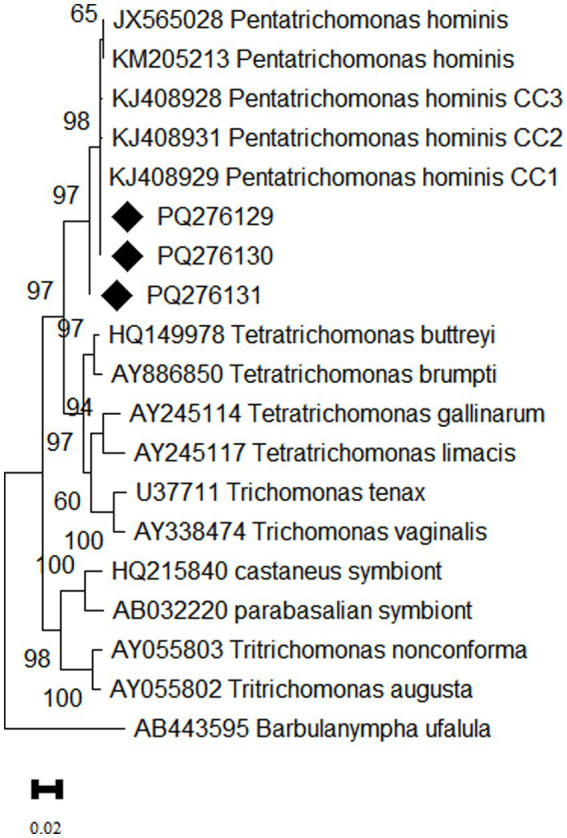
Phylogenetic tree based on the 18S rRNA gene of *P. hominis*. The phylogenetic relationship between *P. hominis* obtained in this study and other known trichomonads were inferred using the maximum likelihood analysis based on the genetic distance calculated by the Kimura 2-parameter model. Bootstrap values more than 50% are shown. The diamond shape denotes isolates from the present study.

Confirmed that the three representative sequences obtained in this study were all classified as *P. hominis*. Among them, sequences PQ276129 and PQ276130 showed 100% homology with the CC1 genotype (KJ408929). However, PQ276131 formed a distinct branch on the phylogenetic tree, indicating that this sequence may represent a novel genetic variant (Figure 3).

## 4 Discussion

Multiple case reports have demonstrated that *P. hominis* is often isolated from patients with diarrhea, suggesting it is a likely cause of gastrointestinal symptoms (6). Furthermore, research indicates that up to 41.54% of gastrointestinal cancer patients are infected with *P. hominis*, which may imply a potential role in the development of gastrointestinal cancer in some cases (22, 23). These findings underscore the importance of not underestimating the potential dangers of *P. hominis*. There is a critical need for further research into its hosts, transmission mechanisms, pathogenicity, and specific role in gastrointestinal diseases. Such studies could lead to the development of effective prevention and treatment strategies for *P. hominis*.

In this study, *P. hominis* was detected in Tibetan antelopes with an infection rate of 1.19% (6/503). This rate is lower compared to the infection rates of *Blastocystis* (4.8%, 30/627) and *Cryptosporidium* (3.0%, 19/627) in Tibetan antelopes (17, 18). The difference may be attributed to the varying survival and transmission capabilities of these parasites in extreme environments. Although Tibetan antelopes are ruminants, their *P. hominis* infection rate is lower than that of dairy cattle (6.8%, 36/526) and beef cattle (4.6%, 15/32), indicating variability in susceptibility among species (13). Notably, the infection rates of *P. hominis* in Siberian tigers (31.3%, 41/131) and silver foxes (43.33%, 26/60) were significantly higher than that in Tibetan antelopes, suggesting that different dietary habits shape distinct gut microbiota, resulting in varying parasite susceptibility (24).

In this study, *P. hominis* was detected in Nima County (6.25%, 4/64) and Shenza County (2.44%, 2/82), while it was not found in the other four counties. Considering the small difference in altitude among the sampling sites, the influence of altitude on the parasite infection rate can be excluded. The differences in infection rates may be related to local ecological and sanitary conditions affecting parasite infection rates. Seasonally, autumn on the Qinghai-Tibet Plateau appears more conducive to *P. hominis* transmission, with an infection rate of 1.42% (6/423) observed in autumn, compared to no infections detected in summer and spring. This may relate to the migratory behavior of Tibetan antelopes, which migrate from April to June and return between August and September (25). Studies suggest that migration not only helps Tibetan antelopes evade predators but may also be a strategy to avoid parasites, thus reducing infection risk in offspring and potentially aiding population growth (26). In conclusion, although no significant effects of seasonal and regional variations on infection rates were observed in this study, the observed trends warrant further investigation with larger sample sizes.

Interestingly, *P. hominis* prevalence was 2% (6/300) in 2020, with no infections detected in 2023 (0/50) and 2024 (0/153). This fluctuation may be linked to global warming, which can alter precipitation patterns and humidity, affecting parasite survival (27). Additionally, changes in vegetation may lead Tibetan antelopes to modify their migration routes and habitats, influencing *P. hominis* infection rates (28). However, the geographical scope of this study is limited, and the sample size is small in some years. Future studies can combine annual sampling with wider geographical coverage and larger sample size to verify this conjecture.

Epidemiological studies have shown that the CC1 genotype was first found in dogs from Changchun, later wildly distribute in *P. hominis* infection in dogs across eastern China. It has also been detected in Siberian tigers, foxes in Henan Province, and primates, including humans in north China, highlighting the strong cross-host transmission ability and potential zoonotic nature of this genotype (1, 7, 10). In this study, two representative *P. hominis* sequences from Tibetan antelopes belonged to the CC1 genotype, indicating that this genotype is the predominant one in Tibetan antelopes, further suggesting that Tibetan antelopes may serve as a potential source of infection of *P. hominis* in humans. Notably, PQ276131 exhibited genetic differences from the CC1, CC2, and CC3 genotypes, indicating that it may represent a novel genotype of *P. hominis*. The newly discovered genotype in Tibetan antelopes may represent a unique adaptation to high-altitude environments, warranting further genetic studies to gain deeper insights into its adaptation mechanisms.

Tibetan antelopes infected with *P. hominis* can easily disperse trophozoites into the environment (29). Given the lifestyle of pastoralists in the Tibetan region, there is a heightened risk of contact with contaminated water or food, increasing the risk of infection. This not only threatens the local ecosystem but also poses potential health risks to residents. Effective measures are needed to monitor and reduce environmental contamination by *P. hominis* and its impact on human health.

## 5 Conclusion

This study is the first to report *P. hominis* infection in Tibetan antelopes. Despite the low prevalence, which may be influenced by sampling and detection methods, it indicates that Tibetan antelopes could be potential vectors of CC1 genotype. This finding provides important data for understanding the epidemiology of *P. hominis* and suggests that Tibetan antelopes should be considered in studies and monitoring of zoonoses in Tibetan areas. Addtionally, this study recommends including other wild animals and livestock in the Tibetan region in the scope of investigation and emphasizes the importance of incorporating *P. hominis* into public health surveillance programs in areas with high human-wildlife interaction.

## Data Availability

The datasets presented in this study can be found in online repositories. The names of the repository/repositories and accession number(s) can be found in the article/supplementary material.

## Footnotes

^1^ http://www.ncbi.nlm.nih.gov/blast/
